# Pet Reptiles: A Potential Source of Transmission of Multidrug-Resistant *Salmonella*

**DOI:** 10.3389/fvets.2020.613718

**Published:** 2021-01-06

**Authors:** Clara Marin, Laura Lorenzo-Rebenaque, Omar Laso, José Villora-Gonzalez, Santiago Vega

**Affiliations:** ^1^Facultad de Veterinaria, Instituto de Ciencias Biomédicas, Universidad Cardenal Herrera-CEU, CEU Universities, Alfara del Patriarca, Spain; ^2^Selvätica Veterinary Clinic, Valencia, Spain

**Keywords:** reptile-associated salmonellosis, multidrug-resistant *Salmonella*, pet reptiles, One Health, zoonosis, *Salmonella*

## Abstract

*Salmonella* spp. is widely considered one of the most important zoonotic pathogens worldwide. The close contact between reptiles and their owners provides favourable conditions for the transmission of zoonotic pathogen infections, and ~6% of human salmonellosis cases are acquired after direct or indirect contact with reptiles. Moreover, antimicrobial resistance is one of the most important health threats of the twenty-first century and has been reported in *Salmonella* strains isolated from pet reptiles, which could entail therapeutic consequences for their owners and breeders. The aim of this study was to assess *Salmonella* carriage by pet reptiles in pet shops and households, and their role in the transmission of antimicrobial resistance, to inform the owners about the possible risks factors. During the period between January 2019 and December 2019, 54 reptiles from pet shops and 69 reptiles from households were sampled in the Valencian Region (Eastern Spain). Three different sample types were collected from each reptile: oral cavity, skin, and cloacal swabs. *Salmonella* identification was based on ISO 6579-1:2017 (Annex D), serotyped in accordance with Kauffman-White-Le-Minor technique, and antibiotic susceptibility was assessed according to Decision 2013/652. The results of this study showed that 48% of the pet reptiles examined from households and pet shops carry *Salmonella* spp. All the strains isolated presented resistance to at least one antibiotic, and 72% were multidrug-resistant strains, the most frequently observed resistance patterns being gentamicin-colistin and gentamicin-colistin-ampicillin. The present study demonstrates that pet reptiles could be a source of human multidrug-resistant *Salmonella* infection. In this context, the most optimal prevention of multidrug-resistant *Salmonella* infections necessarily involves strict control of the sanitary status of reptile pet shops and hygienic handling by the individual owners at home.

## Introduction

*Salmonella* is widely considered one of the most important zoonotic pathogens worldwide. This pathogen has become an important public health concern with a significant economic impact, which has been estimated at 3.6 billion dollars annually (1, 2). In Europe, salmonellosis was responsible for 94,203 human cases, of which 9.3% corresponded to Spain (3). The infection usually causes self-limited diarrhoeal illness, although severe illness and death may occur, especially in children, elderly or immunocompromised adults (4). However, the overall epidemiological pattern of human salmonellosis cases is related to *Salmonella*-contaminated food from animal origin, especially eggs and poultry meat, and ~6% of human salmonellosis cases are acquired after direct or indirect contact with reptiles (3, 4).

In the last few years, exotic reptiles have risen in popularity as pets, with a population of over 7 million in European households (5). This increase in “living presents for children” is resulting in a trade of non-conventional species around the world, with Europe as the leading reptile importer (6, 7). The close contact between reptiles and their owners provides favourable conditions for the transmission of zoonotic pathogens infections, constituting a public health concern, as these pets have been considered as potential *Salmonella* carriers (7–11). Reptiles are natural reservoirs of *Salmonella*, which can hold a wide variety of serovars simultaneously without symptoms (12–14). However, reptile-associated salmonellosis seems to be responsible for more serious complications, with invasive disease and hospitalisation, especially in children (14, 15). From a public health standpoint, pet reptiles represent a persistent source of salmonellosis in households (16–19).

In addition, *Salmonella* multi-resistant strains emerge as a potential concern for public health safety, with implications of increased disease severity, longer hospitalisations and higher cost rates (20, 21). In this context, the World Health Organisation deemed antimicrobial resistance (**AMR**) one of the most important health threats, which could cause 10 million deaths a year by 2050, ahead of other diseases such as cancer (22, 23). In this sense, *Salmonella* has been included in the World Health Organisation priority list of twelve antibiotic-resistant bacteria (24). Interest in the role of reptiles as an antibiotic-resistant *Salmonella* reservoir has increased in recent years (7, 25, 26). Moreover, AMR had been reported in *Salmonella* isolated from captive reptiles, and their release could entail therapeutic consequences for their owners and breeders (16, 27). Moreover, the widespread use of antibiotics against *Salmonella* has been described in the international trade of pet reptiles, in order to prevent economical loses, as well as in animal welfare in crowded farms and long-distance transport (28–30). Therefore, more information on AMR in pet reptiles is needed in view of One Health (31).

In this context, the objective of the present study is to assess *Salmonella* carriage by pet reptiles in pet stores and households in Eastern Spain (Valencia Region) and gain more in-depth knowledge of their role in AMR transmission, in order to inform the owners about the possible risk factors.

## Materials and Methods

All animals were handled according to the principles of animal care published by Spanish Royal Decree 53/2013 (32).

### Sample Collection

During the period between January 2019 and December 2019, a total of 349 samples from 123 different reptile species from households and pet shops reptiles were taken. Previously, the owners were contacted by advertising the project through the University community (Universidad Cardenal Herrera-CEU, and Universidad Politécnica de Valencia) and veterinary clinics of the Valencian Region (Eastern Spain).

A total of 37 species were identified from the 123 of the reptiles sampled (Table 1). From these species, 12 were classified as chelonians (order *Chelonia*), 16 as lizards (suborder *Sauria*) and 9 as snakes (suborder *Ofidia*) (Table 1). According to the individuals sampled from each group, 43.9% (54/123), 39.0% (48/123), and 17.1% (21/123) were chelonians, lizards and snakes, respectively (Table 1).

**Table 1:** *Salmonella* isolated from reptiles in relation to reptile species.

**Category**	**Reptile species**	**Number of reptiles examined**	**Number of positive reptiles (%)**
Suborder *Sauria*	*Zonosaurus ornatus*	1	1 (100)
	*Hemitheconyx caudicinctus*	2	1 (50.0)
	*Correlophus ciliatus*	1	1 (100)
	*Eublepharis macularius*	21	11 (52.4)
	Paroedura picta	2	2 (100)
	*Iguana*	2	2 (100)
	*Tupinambis teguixin*	1	1 (100)
	*Physignathus cocincinus*	5	5 (100)
	*Petrosaurus thalassinus*	2	2 (100)
	*Pogona vitticeps*	4	4 (100)
	*Chamaleo calyptratus*	2	1 (50.0)
	*Varanus glauerti*	1	1 (100)
	*Varanus albigularis*	1	1 (100)
	*Phelsuma grandis*	1	0
	*Pseudopus apodus*	1	0
	*Gecko gecko*	1	0
Suborder *Ofidia*	*Python regius*	5	5 (100)
	*Boa constrictor imperator*	1	1 (100)
	*Gongylophis colubrinus*	1	1 (100)
	*Acrantophis madagascariensis*	1	1 (100)
	*Elaphe guttata*	7	5 (71.4)
	*Spalerosophis diadema*	2	2 (100)
	*Lampropeltis getula*	2	1 (50.0)
	*Basiliscus plumifrons*	1	0
	*Heterodon nasicus*	1	0
Order *Chelonia*	*Graptemys pseudographica*	5	3 (60.0)
	*Testudo marginata*	1	1 (100)
	*Testudo hermanni*	11	5 (45.5)
	*Testudo horsfieldii*	8	1 (12.5)
	*Trachemys scripta elegans*	5	0
	*Pelusios*	1	0
	*Mauremys reevesii*	3	0
	*Cuora flavomarginata*	1	0
	*Pelomedusa subrufa*	1	0
	*Stigmochelis pardalis*	1	0
	*Testudo graeca*	16	0
	*Pseudemys nelsoni*	1	0

For each individual, whenever possible, samples from oral cavity (*n* = 114), skin (*n* = 123), and cloaca (*n* = 112) were taken using sterile cotton swabs (Cary Blair sterile transport swabs, DELTALAB®) (33). All individuals sampled were healthy and none of them presented clinical symptoms such as diarrhoea at the moment of sampling. In addition, an epidemiological questionnaire was filled in. The questionnaire contained information related to species, diet and the number of reptiles that cohabit in the same terrarium. The diet was classified as food from animal origin (including live prey, fresh meat and frozen meat), food of vegetable origin (including fruit and vegetables) and processed (including commercially manufactured reptile food). Moreover, the number of reptiles coexisting in the same terrarium was recorded as reptiles that inhabit alone, or reptiles that cohabit with two or more reptiles.

### Detection of *Salmonella* spp.

The collected samples were analysed within 24 h of collection according to ISO 6579-1:2017 (Annex D) recommendations (34). Samples were pre-enriched in 1:10 vol/vol Buffered Peptone Water 2.5% (BPW; Scharlau, Barcelona, Spain), and then incubated at 37 ± 1°C for 18 ± 2 h. The pre-enriched samples were transferred onto Semi-Solid Modification Rappaport Vassiliadis (MSRV; Difco, Valencia, Spain), and incubated at 41.5 ± 1°C for 24–48 h. For the positive plates, the cultures obtained in MSRV were inoculated onto two specific agar plates for *Salmonella* spp. detection: Xylose Lysine Deoxycholate Agar (XLD; Liofilchem, Valencia, Spain) and a selective chromogenic agar medium specific for detection of C8-esterase activity (ASAP, bioMérieux, Marcy l'Étoile, France), then incubated at 37 ± 1°C for 24 h. After incubation, one typical colony was collected and inoculated into a pre-dried nutrient agar plate (Scharlau, Barcelona, Spain), then incubated at 37 ± 1°C for 24 h. Finally, an API (API-20-E; bioMérieux, Madrid, Spain) biochemical test was performed to confirm *Salmonella* spp. The *Salmonella* isolates were stored at −80°C for further serotyping and antimicrobial susceptibility testing.

### Serotyping and Antimicrobial Susceptibility Testing

From each individual, the serotyping was performed from a cloacal strain, and when not present, a strain from the skin or oral cavity was analysed. Thus, all the strains were unfrozen and revived (ASAP) and the selected isolates were serotyped at the National Reference Laboratory for Animal Health (Algete, Madrid, Spain). The method used for serotyping was antigenic agglutination with specific antisera according to the White-Kauffmann-Le Minor scheme (35).

From all strains, *Salmonella* antimicrobial susceptibility was tested according to the European Committee on Antimicrobial Susceptibility Testing guidelines (36). *Salmonella* strains were inoculated into Mueller-Hinton agar (Scharlab, S.L.) to form a bacterial lawn and were allowed to dry for 30 min at ambient (25°C) temperature; then, the antibiotic discs were applied and plates were incubated at 37°C for 24 h. The antimicrobial agents selected were those set out in Decision 2013/652 (37), including three b-lactams: ampicillin (AMP, 10 μg), cefotaxime (CTX, 30 μg) and ceftazidime (CAZ, 30 μg); two quinolones: ciprofloxacin (CIP, 5 μg) and nalidixic acid (NA, 30 μg); one phenicol: chloramphenicol (CHL, 5 μg); one potentiated sulfonamide: trimethoprim-sulfamethoxazole (SXT, 1.25/23.75 μg); one polymyxin: colistin (COL, 10 μg); one macrolide: azithromycin (AZM, 15 μg); one glycylcycline: tigecycline (TGC, 15 μg); one aminoglycoside: gentamicin (GM, 10 μg); and one pyrimidine: trimethoprim (TM, 5 μg). The source for zone diameters used for interpretation of the test and plates after incubation at 37°C for 24 h was the European Committee on Antimicrobial Susceptibility Testing (EUCAST) (http://www.eucast.org/clinical_breakpoints/), and where this was not possible, according to Clinical and Laboratory Standards Institute (CLSI) indications (https://clsi.org/media/2663/m100ed29_sample.pdf) (38). The isolate strains were categorised as susceptible (S) or resistant (R), based on EUCAST imperative criteria (39). Multidrug resistance (MDR) was defined as acquired resistance to at least one agent in two or more antimicrobial classes (40).

### Statistical Analysis

A Generalised Linear Model, which assumed a binomial distribution for *Salmonella* shedding, AMR and MDR, was fitted to the data to determine whether there was an association with the categorical variables (species and order or suborder of reptile, the habitat of the reptile, sample type, diet and number of reptiles that cohabit in the same terrarium). A reptile was considered *Salmonella* positive if one or more samples collected (oral cavity, skin and/or cloacal) tested positive. A P ≤ 0.05 was considered to indicate a statistically significant difference. Data are presented as least squares means ± standard error of the least squares means. In addition, a descriptive analysis has been done to assess the subespecies isolated in this study. Analyses were carried out using a commercially available software application (SPSS 24.0 software package; SPSS Inc., Chicago, IL, 2002).

## Results

From all samples collected during this study, 25.2 ± 2.3% (88/349) tested positive for *Salmonella*. The type of sample taken was significantly associated with *Salmonella* carriage (*P* = 0.000), with higher positive samples from cloaca (38.0 ± 4.6%, 43/112) than from skin (22.0 ± 3.7%, 27/123) and oral cavity (16.0 ± 3.4%, 18/114).

*Salmonella* spp. was detected in 48.0 ± 4.5% (59/123) of individuals sampled, with significant differences between snakes (76.0 ± 9.3%, 16/21) and lizards (69.0 ± 6.7%, 33/48), compared to chelonians (19.0 ± 5.3%, 10/54) (*P* = 0.000).

The reptiles sampled in this study inhabited households with private owners (56.1%, 69/123), as well as pet shops (43.9%, 54/123) in the Valencian Region (Eastern Spain). Significant differences for *Salmonella* isolation were found among the different reptile habitats (owners vs. pet shops) (*P* = 0.000), being higher in pet shop reptiles (67.0 ± 6.4%, 36/54) than in household pets (33.0 ± 5.7%, 23/69).

Moreover, the number of reptiles cohabiting the same terrarium were known for 111 of the 123 reptiles analysed, 49 for pet shops and 66 for households. In pet shops, significant differences were found between the number of reptiles present in the same terrarium and *Salmonella* shedding (*P* = 0.008). Thus, 89 ± 7.4% of reptiles that cohabit in terrariums with two or more reptiles were positive for *Salmonella* (16/18), while 58 ± 8.9% of reptiles that inhabit terrariums alone were positive for the bacterium (18/31). In contrast, for private owners' reptiles, no significant differences were observed between reptiles that cohabit in terrariums with two or more reptiles or alone and *Salmonella* shedding (*P* = 0.064), 21.0 ± 7.8% (16/38) and 42.0 ± 8.0% (6/28), respectively.

The diet was significantly associated with *Salmonella* carriage (*P* = 0.000), with higher frequency in reptiles that were fed with food from animal origin (65.0 ± 5.6%, 47/72), in contrast to reptiles that were fed with food from vegetable origin, and processed (24.0 ± 6.6%, 10/42, and 22.0 ± 13.9%, 2/9, respectively).

From the 59 strains selected for serotyping, 51 were viable after culture and were serotyped. All *Salmonella* isolates were classified as *Salmonella enterica*. The most represented subspecies were *S. enterica* (56.9%, 29/51), *S. houtenae* (19.6%, 10/51), *S. diarizonae* (11.8%,6/51), *S. salamae* (9.8%, 5/51) and *S. arizonae* (2.0%, 1/51). Fifteen different serovars of *S. enterica* subspecies were identified (Table 2). From all the strains serotyped, one *Salmonella enterica* serovar was indeterminate.

**Table 2:** *Salmonella* serovars isolated from private owners and pet shops.

**Sample origin**	**Subspecies**	**Serovar**	***n***
Private Owner	*enterica*	Albany 8,20:z4,z24	2
		Cerro 18: z4,z23	1
		Lattenkamp 45:z35:1,5	3
		Newport 6,8:e,h:1,2	1
		Paratyphi 4,12:b:1,2	1
	*diarizonae*	60:r:e,n,x,z15	1
		48:z53	1
		50:z52:z35	1
		47:z10:z35	1
		47:i:z53	1
	*arizonae*	44:z4,z23	1
	*houtenae*	11:z4,z23	1
	*salamae*	13, 22:z29:1,5	1
Pet Store	*enterica*	Cotham 28:i:1,5	2
		Fresno 9,46:z38	1
		Hadar 6,8:z10:e,n,x	3
		Hvittingfoss 16:b:e,n,x	1
		Muenster 3,15:e,h:1,5	2
		Newport 6,8:e,h:1,2	2
		Panama 9,12:l,v:1,5	1
		Pomona 28:y:1,7	3
		Sandiego 4,12:e,h:e,n,z15	1
		Vitkin 28:1,v:e,n,x	4
	*houtenae*	11:z4,z23	6
		16:z4,z32	2
		16:z36	1
	*salamae*	30:l,z28:z6	2
		21:g,s,t	1
		52:g,t	1
	*diarizonae*	42:k: z35	1

*n: Number of strains isolated*.

Seventy-five out of 88 *Salmonella* strains isolated were viable after culture and included in the antimicrobial susceptibility study. All strains analysed were resistant to at least one out of the twelve antibiotics tested (*n* = 75/75). The highest percentages of AMR were found to COL (97.3%, *n* = 73), followed by GM (84.0%, *n* = 63), AMP (46.7%, *n* = 35) and TGC (42.7%, *n* = 32), AZM (26.7%, *n* = 20), NAL (12.0%, *n* = 9), CHL (9.3%, *n* = 7), SXT and TM (8.0%, *n* = 6, both), and finally CAZ (6.7%, *n* =5), CTX (4.0%, *n* = 3), and CIP (1.3%, *n* = 1) (*P* = 0.000). Antimicrobial resistance of the different *Salmonella enterica* serovars was summarised in Table 3.

**Table 3:** Percentage of antimicrobial resistance of *Salmonella enterica* isolated from pet reptiles.

***Salmonella enterica* serovars**	***n***	**AMP**	**CTX**	**CAZ**	**CIP**	**NA**	**CHL**	**SXT**	**COL**	**AZM**	**TGC**	**GM**	**TM**
Albany	2	100	0	0	0	100	100	100	100	0	100	100	100
Cerro	1	0	0	0	0	0	0	0	100	0	0	100	0
Cotham	2	100	0	0	0	0	0	0	100	0	100	100	0
Fresno	1	0	0	0	0	0	0	0	100	0	0	100	0
Hadar	3	66.7	0	0	0	0	0	0	100	100	66.7	100	0
Hvittingfoss	1	100	0	0	0	0	0	0	100	0	100	100	0
Lattenkamp	3	0	0	0	0	0	0	0	66.7	0	0	33.3	0
Muenster	2	100	0	0	0	0	0	0	100	50	50	100	0
Newport	3	0	0	0	0	0	0	0	100	0	33.3	66.7	0
Panama	1	0	0	0	100	0	0	0	100	0	0	100	0
Paratyphi	1	0	0	0	0	0	0	0	100	0	0	100	0
Pomona	3	100	0	33.3	0	0	0	0	100	66.7	0	100	0
Sandiego	1	100	0	0	0	0	0	0	100	100	100	100	0
Vitkin	4	50	0	25	0	0	0	0	100	0	75	75	0

*n: Number of samples. The resistance was determined by disc diffusion. AMP, Ampicillin (10 μg); CTX, Cefotaxime (30 μg); CAZ, Ceftazidime (30 μg); CIP, Ciprofloxacin (5 μg); NA, Nalidixic acid (30 μg); CHL, Chloramphenicol (5 μg); SXT, Trimethoprim-sulfamethoxazole (1.25/23.75 μg); COL, Colistin (10 μg); AZM, Azithromycin (15 μg); TGC, Tigecycline (15 μg); GM, Gentamicin (10 μg); TM, Trimethoprim (5 μg)*.

Furthermore, a total of 72.0% (54/75) *Salmonella* isolates were resistant to two or more antimicrobials. No significant differences in MDR rates were shown between lizards (78.0%, 32/41), chelonians (73.3%, 11/15) and snakes (57.9%, 11/19) (*P* = 0.206). Although the type of sample collected was not significantly associated with MDR carriage, oral cavity (75.0%, 12/16), skin (77.3%, 17/22) and cloacal samples (67,6%, 25/37) (*P* = 0.692), significant differences were found between the type of sample and the different types of reptiles, except for lizards (Table 4, *P* < 0.05). Moreover, no significant differences were found between the habitat (pet shop and household), diet (food from animal origin, vegetable origin, and processed) and MDR *Salmonella* carriage (*P* = 0.065 and *P* = 0.432, respectively)

**Table 4:** Multidrug-resistant *Salmonella* isolated according to the type of sample collected in the different type of reptiles.

**Reptile classification**	**Type of sample**	***n***	**MDR rate**
Suborder *Sauria*	Oral cavity	9	89.0 ± 10.5
	Skin	13	79.0 ± 9.4
	Cloacal	19	69.0 ± 12.8
Suborder *Ofidia*	Oral cavity	3	0.0 ± 0.0^a^
	Skin	23	100.0± 0.0^b^
	Cloacal	13	62.0 ± 13.5^c^
Order *Chelonia*	Oral cavity	4	100.0± 0.0^a^
	Skin	6	83.0 ± 15.2^a^^b^
	Cloacal	5	40.0 ± 21.9^b^

*Data are presented as least squares means ± standard error of the least squares means*.

a,b,c *Different superscripts in each file means significant differences in the same reptiles' classification with a P < 0.05. MDR, Multidrug resistance. n, Number of samples*.

Overall, 25 different resistance patterns were observed. The combination of GM-COL (18.7%, 14/75) was the most frequently observed pattern, followed by GM-COL-AMP and GM-COL-TGC (10.7%, 8/75, both), COL alone (9.3%, 7/75) and GM-COL-AMP-TGC (8.0%, 6/75).

## Discussion

The present study demonstrates that 48% of the pet reptiles examined from households and pet shops carry *Salmonella* spp. All the strains isolated showed resistance to at least one antibiotic and 72% were multidrug-resistant strains. To our knowledge, this is the first study in the literature evaluating the prevalence and the antimicrobial resistance of this zoonotic pathogen from a considerable sample size in pet reptiles of Eastern Spain (Valencia Region).

Reptiles have been known to be important carriers of *Salmonella* spp. worldwide, which may pose a health hazard as a source of human infection, particularly in children (4, 16, 41–43). However, there is a lack of consensus regarding the role of reptile shops on MDR *Salmonella* strains spreading. The results of this study showed that *Salmonella* strains isolated in reptiles from shops were twice as high as those from private owners (67 vs. 33%) (44, 45). This may be due to poor hygienic management of terrariums, especially in pet shops where they are usually occupied and ensuring a proper cleaning and disinfection procedure is not easy (45). This fact could facilitate that MDR strains remain in the shop environment among different reptile batches. In addition, the reptiles' stress related to cohabiting with individuals of different ages and origins could result in an increase in the bacterial infection, shedding in the terrarium and reptile-to-reptile transmission (45, 46). Conversely, reptiles from private owners are exposed to better hygiene practises and less stressful environments, leading to lower *Salmonella* shedding (45).

In reptiles, *Salmonella* is spread by faecal-oral route with an asymptomatic natural colonisation of the enteric tract, so in this study cloacal swabs collected were more sensitive for *Salmonella* isolation than other samples collected, such as skin or oral cavity. However, it is important to highlight that because *Salmonella* is excreted through faeces, it could contaminate the reptile's skin, oral cavity and the environment, being a source of infection for humans who handle the reptile or who are exposed to the reptile's environment (11, 46–50). Moreover, the *Salmonella* serovars most frequently detected in this study have been cited previously in reptile studies (51, 52), as well as in human outbreaks (3, 51–55). In addition, it has been reported that cold-blooded animals could be the major reservoir for the subspecies *houtenae, diarizonae, salamae*, and *arizonae* (56, 57).

The results of this study showed higher *Salmonella* prevalence among snakes and lizards compared to chelonians, in accordance with previous research (11, 31, 52, 58, 59). Particular attention has recently been given to snakes and lizards, as human interaction with these reptiles has become increasingly common in domestic environments (11, 60). In this sense, it is important to highlight that these reptiles are mainly fed with food from animal origin, which represents an important source of *Salmonella* (49, 61, 62). Previous studies carried out in the United Kingdom reported the important role of commercial feeder rodents in bacterial transmission among reptiles, and even their owners (63, 64). Thus, handling *Salmonella*-contaminated feeder rodents, as well as cross-contamination in the kitchen due to the rodents being kept in the freezer and thawed in microwaves, also in contact with food for human consumption, have been linked to human *Salmonella* outbreaks (63, 65). In this context, to avoid *Salmonella* infection of reptiles, control of food products of animal origin has to be mandatory for the food suppliers (64).

On the other hand, special attention must be given to chelonians, due to their popularity as a pet for children. In this context, several countries, such as the US, have implemented strict bans in an attempt to curtail chelonian-associated salmonellosis; however, in Europe there are not many regulations to control its prevalence (53, 66). In the present study, *Salmonella* has been isolated from 18.5% of the chelonians tested. Seasonal effects, such as hibernation or season of sampling, have been speculated by previous studies to explain the low isolation rate of *Salmonella* in chelonians compared to other reptiles (31). Moreover, the diet may also have an important role (43, 49) because, as reported above, a large proportion of the chelonians are fed with food from vegetable origin or processed, and not from animal origin, frequently related with *Salmonella* outbreaks (11, 31, 67).

The increase in MDR *Salmonella* strains is of worldwide interest because it enhances the risk of therapeutic failure in cases of life-threatening salmonellosis in human and veterinary medicine (68, 69). In fact, it has been estimated that AMR could be the main cause of human mortality in 2050 (22). One of the most relevant outcomes in this study was the level of MDR isolated from pet reptiles, the most frequently observed resistance patterns being GM-COL and GM-COL-AMP. The high resistance against GM could be explained due to the indiscriminate use of aminoglycosides in pet reptile breeders, especially in the chelonian industry (28, 70). The use of GM as prophylactic *Salmonella* treatment in eggs to ensure sanitary conditions is a common practise in the US, the main supplier country of live reptiles for the EU (28, 71). Indeed, this practise has contributed to the finding of high-level plasmid-mediated gentamicin resistance in *Salmonella* isolated in its breeder farms (28).

Polymyxins have been widely used against Gramme-negative infections in animals, especially in animal production, the origin of several products involved in reptile feeding (72, 73). Currently, polymyxins such as COL represent the last line of defence against severe resistant infections in humans (72). Thus, it is highly restricted for animal infection treatments, and it is expected that resistance to this antibiotic will decrease in the coming years (74–76).

AMP was the third most frequent resistance shown in this study of *Salmonella* reptile strains, in line with a previous study conducted on species of gecko in Italy (10). This antibiotic is the most widely used in human medicine in Spain (76) and could thus be implicated in possible transmission of resistance from humans to reptiles, as a consequence of the direct and indirect contact between reptiles and their owners (75, 77, 78).

On the other hand, the level of resistance to NAL, CHL, SXT, TM, CAZ, CTX, and CIP in reptile *Salmonella* strains was relatively low. Resistance to CAZ contrasts with a survey conducted on geckos by Russo et al. (10), who showed a high resistance of *Salmonella* species to this antimicrobial, that is used in reptiles to treat infections or prophylactically after a traumatic injury (79). Moreover, it is important to note that fluoroquinolones (e.g., CIP) are the drugs of choice for invasive salmonellosis infections in humans (adults) and cephalosporins (e.g., CTX and CAZ) in children (21), although both are implicated in a reduced effectiveness of *Salmonella* treatment (77).

Should be essential to inform pet reptile owners about the risks of wrongly handling these animals (60). Proper hygienic management measures should be taken, such as the use of gloves when cleaning the reptile, and even during cleaning and disinfection of the surfaces that come in contact with the pet reptile (13). Moreover, it is important to thorough hand washing after handling the reptiles, especially the wounds due to bites or scratch (13). Reptiles and its feed should keep away from the kitchen and areas where the owners prepare their own food (64). Besides, before introducing a new reptile in the household, microbiological exams should be carried out to avoid cross-infection (13). Finally, it highlights the importance of extreme caution with young children and immunocompromised patients, because they are especially susceptible to *Salmonella* spp. infections (60).

## Conclusions

The present study clearly demonstrates that pet reptiles could be a source of human MDR *Salmonella* infection. The problem of MDR in reptiles could start with the shops, where *Salmonella* presence is extremely high, and seems to be linked with the origin of reptile food. In this context, the most optimal prevention of MDR *Salmonella* infections involves strict control of the sanitary status of reptile pet shops and hygienic handling of the individuals in the household. Nevertheless, it is important to highlight that the number of included samples is relatively small, which may restrict the interpretation of our results to Eastern Spain. Further studies are needed to validate our results in a larger study sample.

## Data Availability Statement

The raw data supporting the conclusions of this article will be made available by the authors, without undue reservation.

## Ethics Statement

Ethical review and approval was not required for the animal study because All animals were handled according to the principles of animal care published by Spanish Royal Decree 53/2013 (32). Written informed consent was obtained from the owners for the participation of their animals in this study.

## Author Contributions

CM, OL, and SV: conceptualisation. OL, JV-G, CM, and SV: data curation. LL-R, CM, OL, and JV-G: methodology. CM and SV: investigation, writing–review and editing, and funding acquisition. CM and LL-R: writing–original draft preparation. SV: project administration. All authors: have read and agreed to the published version of the manuscript.

## Conflict of Interest

The authors declare that the research was conducted in the absence of any commercial or financial relationships that could be construed as a potential conflict of interest.
